# Effects of the SLC38A2–mTOR Pathway Involved in Regulating the Different Compositions of Dietary Essential Amino Acids–Lysine and Methionine on Growth and Muscle Quality in Rabbits

**DOI:** 10.3390/ani12233406

**Published:** 2022-12-03

**Authors:** Bin Zhang, Boyuan Ning, Xiaoyang Chen, Chenyang Li, Mengqi Liu, Zhengkai Yue, Lei Liu, Fuchang Li

**Affiliations:** 1 Key Laboratory of Efficient Utilization of Non-Grain Feed Resources (Co-Construction by Ministry and Province), Ministry of Agriculture and Rural Affairs, Shandong Provincial Key Laboratory of Animal Biotechnology and Disease Control and Prevention, Department of Animal Science, Shandong Agricultural University, Taian 271018, China; 2 State Key Laboratory of Animal Nutrition, Institute of Animal Science, Chinese Academy of Agricultural Sciences, Beijing 100193, China; 3 College of Animal Science and Technology, Northwest A&F University, Xianyang 712100, China; 4 College of Animal Science and Technology, China Agricultural University, Beijing 100083, China

**Keywords:** SLC38A2–mTOR pathway, amino acid metabolism, dietary lysine and methionine composition, rabbits, muscle growth and quality, nitrogen metabolism

## Abstract

**Simple Summary:**

China is not only a huge meat rabbit consumer but also the largest meat rabbit producer in the world, contributing a large amount of rabbit meat products to the domestic and foreign markets every year. Therefore, it is important for the domestic and international rabbit meat market to improve rabbit breeding production efficiency and rabbit meat quality based on the good use of domestic feed resources in China. It is well known that dietary amino acid nutrition is of great importance to animal growth. Lysine and methionine are limited in the common domestic rabbit feed sources in China, but they play an important role in rabbit growth. Moreover, different lysine and methionine compositions of the diets respond differently to rabbit growth. Consequently, the search for a better composition of dietary lysine and methionine is the main objective of this study.

**Abstract:**

In recent years, ensuring food security has been an important challenge for the world. It is important to make good use of China’s domestic local feed resources to provide safe, stable, efficient, and high-quality rabbit meat products for China and the world. Lysine and methionine are the two most limiting essential amino acids in the rabbit diet. However, little is known about the rational composition of lysine and methionine in rabbit diets and the mechanisms that affect growth and development. Accordingly, in this study, we sought to address this knowledge gap by examining the effects of different compositions of lysine and methionine in rabbit diets. Subsequently, the growth status, nitrogen metabolism, blood biochemical indexes, muscle development, muscle quality, and the growth of satellite cells were evaluated in the animals. The results showed that diets containing 0.80% Lys and 0.40% Met improved average daily weight gain, feed conversion, nitrogen use efficiency, and muscle quality in the rabbits (*p* < 0.05). Additionally, it altered the amino acid transport potential in muscle by upregulating the expression of the *SLC7A10* gene (*p* < 0.05). Meanwhile, the cell viability and the rate of division and migration of SCs in the 0.80% Lys/0.40 % Met composition group were increased (*p* < 0.05). SLC38A2 and P–mTOR protein expression was upregulated in the 0.80% lysine/0.40% methionine composition group (*p* < 0.05). In conclusion, 0.80% Lys/0.40% Met was the most suitable lysine and methionine composition in all tested diets. SLC38A2 acted as an amino acid sensor upstream of mTOR and was involved in the 0.80% Lys/0.40% Met regulation of muscle growth and development, thus implicating the mTOR signaling pathway in these processes.

## 1. Introduction

Amino acids are of great physiological importance, serving as the building blocks for proteins, as well as substrates for the synthesis of low–molecular–weight substances [1]. These biomolecules have traditionally been classified as nutritionally “essential” or “non-essential” based on the growth or nitrogen balance of animals [2]. The carbon skeleton of essential amino acids cannot be synthesized de novo by animal cells, and these amino acids must be obtained from the diet to sustain life. In contrast, nutritionally non-essential amino acids can be synthesized de novo in sufficient amounts within cells, and are normally considered dispensable in the diet [3]. However, the nitrogen balance is not a sensitive indicator of optimal dietary amino acid requirements [4]. Most amino acids also function as signaling molecules in the regulation of animal metabolism, and thus their levels must be fine-tuned to meet a variety of important needs, such as energy balance, protein synthesis, and cell and tissue development [5].

Lysine (Lys) is the most limiting essential amino acid in mammalian grain diets and is believed to promote the growth of muscle fibers in vertebrate skeletal muscle through the stimulation of protein synthesis [6,7]. Lys deficiency can result in significant physical growth restriction and weight loss [8]. The important role of Lys in promoting skeletal muscle growth has been demonstrated in animal husbandry and is attributable to increased protein synthesis [9]. Methionine (Met) is an essential amino acid in mammals. In addition to being a component of proteins, Met also plays a role in many important metabolic and non-metabolic pathways, including epigenetics (S–adenosylmethionine synthesis), nuclear activity (polyamine production), detoxification (as a constituent of glutathione), and the methylation of cell membrane phospholipids (regulation of cell metabolism) [10]. Moreover, the Met cycle is closely related to folic acid metabolism, thereby indirectly regulating nucleotide biosynthesis [11].

The supplementation of limiting essential amino acids for protein synthesis has long been thought to increase weight gain and muscle mass via an unknown molecular pathway [12]. In addition to being substrates for protein synthesis, amino acids are also nutritional signals and regulators of protein metabolism, for example, by regulating the functions of translation initiation factors and elongation factors [13]. Neutral aliphatic amino acids, including Met and branched–chain amino acids, reportedly stimulate the phosphorylation of ribosomal protein S6 kinase, a downstream target of the mammalian target of rapamycin (mTOR) signaling pathway, thus promoting protein synthesis [14]. Meanwhile, Lys regulates skeletal muscle growth and inhibits myotube protein degradation by activating the mTOR pathway in skeletal muscle [15]. The mTOR pathway has been shown to play an important role in the activity of satellite cells (SCs), especially their division and proliferation [16]. SCs are skeletal muscle stem cells important for the maintenance of the morphological and functional stability of muscle fibers. Their ability for self-renewal and proliferation not only helps maintain the muscle stem cell pool but also represents a source of abundant muscle-derived cells. The proliferation, differentiation, and fusion of SCs lead to the formation of new muscle fibers and the reconstruction of functional contractile devices [17].

Lys and Met are the two most limiting essential amino acids in the rabbit diet. However, little is known about the effects of dietary lysine and methionine composition on rabbit growth and development. This study was undertaken to evaluate the effects of different dietary Lys and Met compositions on muscle growth and development in rabbits. For this, the growth status (feed intake, body weight, and survival rate), nitrogen metabolism, blood biochemistry, and muscle quality were evaluated. The effects of Lys and Met composition on the expression levels of relevant target genes and proteins in tissues and SCs were also assessed. Our findings provide not only novel insights into the formulation of rabbit diets for the improvement of meat quality and the exploration of the underlying mechanisms but also an important reference for future dietary amino acid utilization in the diets of rabbits and other animals.

## 2. Materials and Methods

### 2.1. Animal Housing and Diets

The rabbit house was naturally ventilated and illuminated, with a temperature of approximately 28 °C at noon and 20 °C at night (May in Tai’an, China). Five rabbits were kept inside a cage (200 cm × 200 cm × 100 cm) and shared feed and water (free feeding and watering). Cages had open cage tops and food-grade rigid plastic floors. The basic feed was formulated according to the NRC (National Research Council) (1977) Nutritional Requirement of Rabbits guidelines and Nutrition of the rabbit [18]. The test diets containing different compositions of lysine and methionine were formed by adding different levels of lysine and methionine to the levels of lysine and methionine contained in the basal diet. The composition and nutrient levels of the basic diet are shown in Supplementary Table S1 while the amounts of Lys and Met added to the experimental diet are shown in Supplementary Table S2.

Experiment 1:240 male Hyla rabbits (35 days old) with similar body weight (1100 ± 10 g) were divided into eight groups (6 replicates per group, with 5 rabbits per replicate). Eight levels of Lys and Met composition (0.75% Lys/0.10% Met, 0.75% Lys/0.25% Met, 0.75% Lys/0.50% Met, 0.75% Lys/0.75% Met, 0.60% Lys/0.40% Met, 0.80% Lys/0.40% Met, 1.00% Lys/0.40% Met, and 1.20% Lys/0.40% Met) were selected for testing. According to the feed intake, daily weight gain, and health status of the rabbits after 10 days of the test (Figure S1), the 0.75% Lys/0.25% Met, 0.75% Lys/0.50% Met, 0.80% Lys/0.40% Met, and 1.00% Lys/0.40% Met composition levels were selected for use in Experiment 2.

Experiment 2: A total of 120 male Hyla rabbits (35 days old) with similar body weight (1100 ± 10 g) were divided into four groups (6 replicates per group, with 5 rabbits per replicate) and fed experimental diets containing the above-mentioned Lys and Met composition. At the beginning of the experiment, all the rabbits were weighed and the feed intake of each group was determined once every 5 days. After 40 days (75 days old), the weight, feed intake, and health status of the rabbits were assessed. At the end of the experiment, six rabbits in each group (each duplicate selected one, the same below) were selected (a rabbit whose weight was the average weight of each replicate) for blood collection. Blood was collected with a syringe from ear veins of the animals and transferred to vacuum blood collection tubes containing an anticoagulant. After centrifugation at 3000 rpm for 10 min, the supernatant was collected and stored at −80 °C. The 24 rabbits were then euthanized by cervical dislocation, and samples (liver, kidney, muscle) were collected, weighed, snap–frozen in liquid nitrogen, and stored at −80 °C.

### 2.2. Detection of Nitrogen Metabolism and Feed Conversion Ratio

According to previous studies by Chen et al. [19], during the last 3 days of the experiment, six rabbits in each group were randomly selected for the once-daily collection of feces and urine. After weighing, the collected samples were fixed in 10% sulfuric acid and stored at −80 °C for subsequent testing. The nitrogen content in the samples was detected using a Kjeldahl Nitrogen Analyzer (FOSS, Hilleroed, Denmark). Nitrogen-related parameters were calculated using the following formulae: Digestible nitrogen (g/day) = ingested nitrogen–fecal nitrogen; Deposition of nitrogen (g/day) = ingested nitrogen–fecal nitrogen–urinary nitrogen; Apparent digestibility of nitrogen (%) = digestible nitrogen/ingested nitrogen × 100%; Nitrogen utilization rate (%) = nitrogen deposition/ingested nitrogen × 100%; Nitrogen biological titer (%) = nitrogen deposition/digestible nitrogen × 100%; Feed conversion ratio (%) = average daily gain/average daily feed intake × 100%.

### 2.3. Quantitative Real–Time PCR (RT–qPCR)

Total RNA extraction was performed as previously described [20]. The quality and quantity of extracted RNA were determined using agarose gel electrophoresis and a biophotometer (Eppendorf, Hamburg, Germany), respectively. Primers targeting exon–intron junctions were designed using Primer 6.0 software (Primer–E Ltd., Plymouth, UK). The primer sequences are shown in Table S3. RT–qPCR was performed according to the method described in Accurate Biology SYBR^®^ Green Premix Pro Taq HS qPCR Kit (AG11718, Accurate Biology, Hunan, China). Relative gene expression levels were calculated using the 2–∆∆CT method after normalization to the levels of the glyceraldehyde 3–phosphate dehydrogenase (GAPDH) and the *β*–actin genes. Based on the cycle threshold (CT) values, GAPDH and *β* –actin mRNA expression was stable across treatments in this study (*p* > 0.1).

### 2.4. SLC38A2 Knockout

Determine the screening concentration of puromycin: Cells were tested for sensitivity to puromycin at concentrations such as 0, 0.2, 0.5, 1, 1.5, 2, 3, 4, and 5 µg/mL. The lowest concentration at which all cells die after two days was the puromycin screening concentration for that cell. SCs were inoculated into 6–well plates and cultured with cell numbers for the next day to reach 50% cell fusion. After incubation at 37 °C overnight, 3 μg/mL of puromycin was added to the culture medium. The cells were infected by Beyotime’s SLC38A2 knockout lentivirus product (L23166, Beyotime, Shanghai, China). After two days, the medium containing the virus was aspirated, and medium containing puromycin was added. After two days of incubation at 37 °C, live cells were collected and assayed for SLC38A2 protein expression.

### 2.5. Western Blotting

Total protein was extracted from skeletal muscle SCs using RIPA(Radio immunoprecipitation assay) lysis buffer containing the protease inhibitor PMSF. Protein concentration was measured using the BCA (Bicinchoninic acid) Protein Assay Kit (Thermo Fisher, Waltham, MA, USA) after centrifugation at 12,000 rpm for 15 min at 4 °C. A total of 10 µg of protein was separated by 8–10% sodium dodecyl sulfate–polyacrylamide gel electrophoresis (SDS–PAGE), transferred to polyvinylidene fluoride membranes (Millipore, Darmstadt, Germany), blocked for 1 h, and then incubated with primary antibody overnight at 4 °C. After four 10 min washes, the membrane was incubated with the secondary antibody for 1 h and then washed again four times, 10 min each wash. Immunoreactivity was detected using an enhanced chemiluminescence (ECL) kit (P2300, NCM Biotech, Suzhou, China) and visualized using the Fluor Chem M system. Image J v2 software was used for quantitative analysis.

### 2.6. Supplementary Materials and Methods

The methods used for detection of detection of muscle quality, plasma biochemistry, SCs isolation and culture, the cell migration assay, SCs identification (Figure S2), immunofluorescence, the cell cycle and apoptosis assays and mTOR pathway activation and inhibition assays are described in Supplementary Materials.

### 2.7. Statistical Analysis

Data were analyzed by a one-factor general linear model (GLM) using the SAS v9.2 software package (SAS Inst. Inc., Cary, NC, USA). Duncan’s multiple range test was used to indicate the significance of differences at *p* < 0.05. Data were expressed as means ± SEM. Means were considered to be significantly different when *p* < 0.05 and a tendency when 0.05 ≤ *p* ≤ 0.10.

## 3. Results

### 3.1. Effects of Different Lys and Met Composition in Diets on the Growth and Body Metabolism of Rabbits

As shown in Table 1, the average daily feed intake of rabbits was highest when the diet contained 0.75% Lys and 0.25% Met; the average daily feed intake was lowest when the diets contained 0.10% and 0.40% Met (*p* < 0.05). However, the average daily gain was highest with the 0.80% Lys/0.40% Met composition (*p* < 0.05). Among the four experimental groups, the highest feed conversion was observed in the group containing 0.80% Lys/0.40% Met, and the lowest feed conversion was observed in the group containing 0.75% Lys/0.25% Met (*p* < 0.05). No significant differences in liver weight were detected among the four diets (*p* > 0.05). However, the 0.75% Lys/0.25% Met composition group had the lowest kidney weight, with significant differences compared to the 0.75% Lys/0.50% Met composition group (*p* < 0.05).

By examining the effect of different lysine and methionine composition of diets on nitrogen metabolism in rabbits, we found that none of the four test diets exerted significant effects on nitrogen intake, digestible nitrogen, and retention of nitrogen by the rabbits (*p* > 0.05, Table 2). Among the four test groups, the 0.80% Lys/0.40% Met composition group had the lowest fecal and urinary nitrogen content (*p* < 0.05, Table 2). Nitrogen apparent digestibility, nitrogen utilization, and nitrogen biological value were significantly higher in rabbits provided with 0.80% Lys/0.40% Met in the other diet than in those provided with the 0.75% Lys/0.25% Met composition (*p* < 0.05, Table 2). Meanwhile, after blood biochemical tests in rabbits, we found plasma uric acid and urea were lowest in the 0.80% Lys/0.40% Met composition group, but differed significantly only from the 1.00% Lys/0.40% Met composition group (*p* < 0.05, Table 3); however, albumin, glucose, total cholesterol, triglyceride, and total protein levels did not differ significantly among the four test groups (*p* > 0.05, Table 3).

### 3.2. Effects of Diets with Different Lys and Met Composition on Muscle Traits and Gene Expression in Rabbits

**Figure 1:**
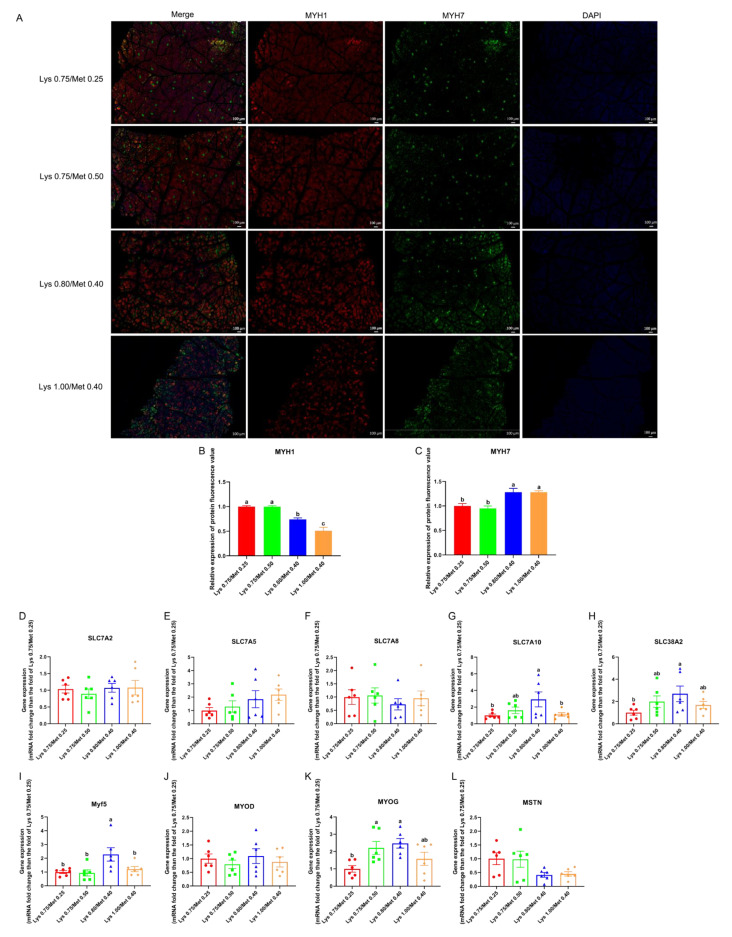
Effects of different lysine and methionine composition in diets on muscle fiber type and muscle tissue target gene expression in rabbits. (**A**–**C**) Muscle type detection (muscle tissue immunofluorescence). (**A**) MYH1 (red fluorescent, myosin–1; rabbit polyclonal fast myosin skeletal heavy chain antibody) and MYH7 (green fluorescent, myosin–7; rabbit polyclonal slow skeletal myosin heavy chain antibody). Quantification of MYH1 (**B**) fluorescence and MYH7 (**C**) fluorescence. (**D**–**H**): the expression of amino acid transporter protein-encoding genes. *SLC7A2* (D), *SLC7A5* (E), *SLC7A8* (F), *SLC7A10* (**G**), and *SLC38A2* (H). (**I**–**L**): the expression of genes encoding muscle development-related regulatory factors. *MYF5* (I), *MYOD* (**J**), *MYOG* (**K**), and *MSTN* (**L**). Data are expressed as means ± SEM (*n* = 6). Comparisons between groups that contain only different lowercase letters indicate significant differences (*p* < 0.05).

In the four experimental groups, we detected changes in muscle tissue fiber types in rabbits by immunofluorescence, and we found MYH1 had the highest protein expression in the groups providing 0.75% Lys/0.25% Met and 0.75% Lys/0.50% Met composition, followed by the 0.80% Lys/0.40% Met composition group, and the lowest was the 1.00% Lys/0.40% Met composition group (*p* < 0.05, Figure 1A,B). However, MYH7 protein expression was significantly downregulated in the 0.75% Lys/0.25% Met and 0.75% Lys/0.50% Met composition groups compared with that in the other two groups (*p* < 0.05, Figure 1A,C).

Through testing of other muscle quality indicators, we found that muscle shear force was greatest in the 0.75% Lys/0.25% Met composition group, followed by the 0.80% Lys/0.40% Met composition group; the smallest muscle shear force was seen in the group provided with 1.00% Lys/0.40% Met composition (*p* < 0.05, Table 4). The greatest drip loss was observed in the group administered the 0.75% Lys/0.50% Met composition, with the lowest being recorded with 1.00% Lys/0.40% Met composition (*p* < 0.05, Table 4). At 45 min post-euthanasia, the 0.80% Lys/0.40% Met composition group exhibited the lowest muscle pH values and the 0.75% Lys/0.25% Met composition group the highest (*p* < 0.05, Table 4). However, 24 h after euthanasia, muscle pH values were not significantly different among the four groups of rabbits (*p* > 0.05, Table 4). Similarly, no significant changes in flesh color (a *, b *, L *) were observed among the groups (*p* > 0.05, Table 4).

Further, by RT–qPCR assays of target genes related to muscle tissue development, we identified the highest *SLC7A10* gene expression level was found in the 0.80% Lys/0.40% Met composition group, with significant upregulation being observed relative to the 0.75% Lys/0.25% Met and 1.00% Lys/0.40% Met composition groups (*p* < 0.05, Figure 1G). Similarly, *SLC38A2* gene expression was also increased in the group administered the 0.80% Lys/0.40% Met composition relative to that in the other three groups, reaching significance compared with the 0.75% Lys/0.25% Met composition group (*p* < 0.05, Figure 1H). The expression of the *Myf5* gene was significantly higher in the 0.80% Lys/0.40% Met composition group than in the other three groups (*p* < 0.05, Figure 1I). Meanwhile, *MYOG* gene expression was also highest in the 0.80% Lys/0.40% Met composition group, and differed significantly from that seen in the group administered 0.75% and 0.25% Met in the diet (*p* < 0.05, Figure 1K). However, the transcript levels of *SLC7A2*, *SLC7A5*, *SLC7A8*, *MYOD*, and *MSTN* did not differ significantly among the four experimental groups (*p* > 0.05, Figure 1D–F,J,L).

### 3.3. Effects of Different Lysine and Methionine Composition in Diets on the Growth of Rabbit SCs

**Figure 2:**
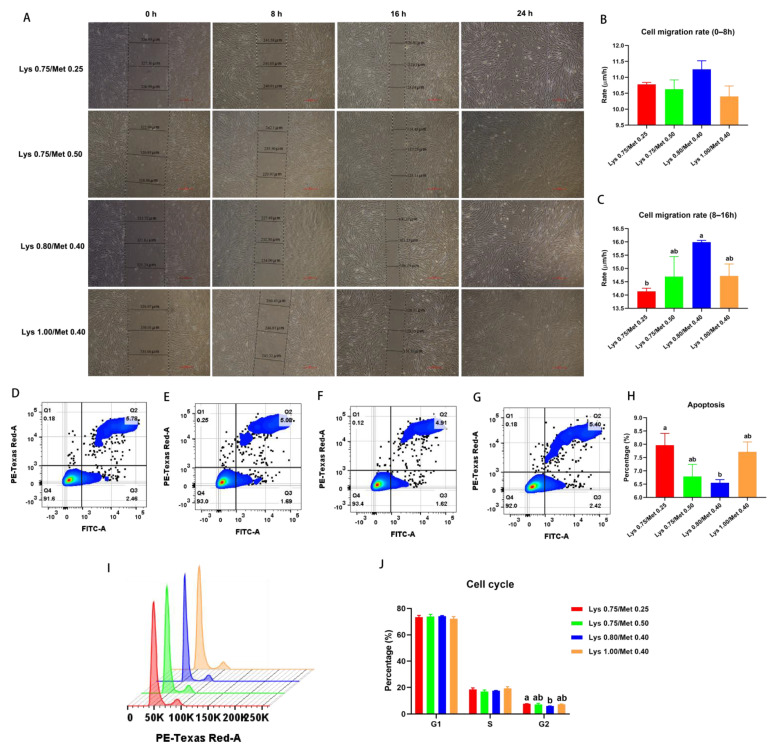
Effects of different dietary lysine and methionine composition on satellite cell migratory potential, division, and apoptosis in rabbits. (**A**–**C**) Cell migration assay. Image recording of cell migration (**A**), cell migration rate between 0 and 8 h (**B**), and cell migration rate between 8 and 16 h (**C**). (**D**–**H**) Cell apoptosis. (**D**) 0.75% Lys/0.25% Met composition group, (**E**) 0.75% Lys/0.50% Met composition group, (**F**) 0.80% Lys/0.40% Met composition group, (**G**) 1.00% Lys/0.40% Met composition group, and (**H**) statistics for cell apoptosis data. Cell cycle flow assay (**I**), cell cycle statistics (**J**). Data are expressed as means ± SEM (*n* = 3). Comparisons between groups that contain only different lowercase letters indicate significant differences (*p* < 0.05).

To further determine the mechanism of the effect of different lysine and methionine composition of diets on muscle growth and development, we conducted an in vitro experiment with rabbit muscle satellite cells. As shown in Figure 2, there was no significant difference in the SCs migration rate among the four experimental groups from 0 to 8 h (*p* > 0.05, Figure 2A,B). From 8 to 16 h, the 0.80% Lys/0.40% Met composition group exhibited the highest cell migration rate, reaching significance compared with the 0.75% Lys/0.25% Met composition group (*p* < 0.05, Figure 2A,C). Additionally, we found that the 0.80% Lys/0.40% Met composition group displayed the lowest proportion of apoptotic cells among the four groups, with a significant difference being noted relative to the 0.75% Lys/0.25% Met composition group (*p* < 0.05, Figure 2D–H). Similarly, the 0.80% Lys/0.40% Met composition group exhibited the smallest percentage of cells in the G2 phase of the cell cycle, and differed significantly when compared with the group treated with the 0.75% Lys/0.25% Met composition level (*p* < 0.05, Figure 2I,J). The numbers of cells in the G1 and S phases were not significantly different among the four test groups (*p* > 0.05, Figure 2I,J).

### 3.4. Effects of Different Lys and Met Composition on the mTOR Signaling Pathway in Muscle Tissue and SCs

**Figure 3:**
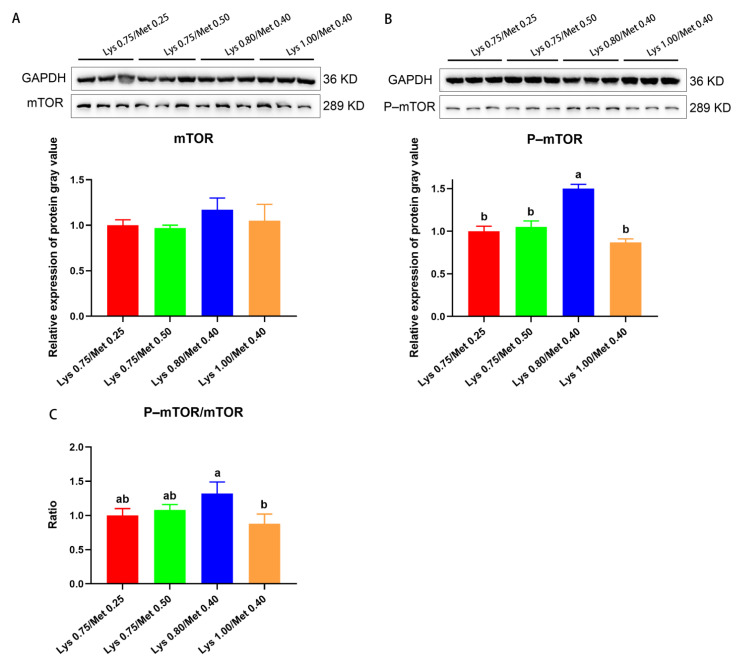
Effects of lysine and methionine composition in the diet on the mTOR signaling pathway in rabbit muscle. (**A**–**C**) Detection of the mTOR signaling pathway in rabbit muscle. Relative mTOR protein expression in muscle (**A**), relative levels of phosphorylated mTOR protein (P–mTOR) in muscle (**B**), and the P–mTOR/mTOR ratio (**C**). Data are expressed as means ± SEM (n = 3). Comparisons between groups that contain only different lowercase letters indicate significant differences (*p* < 0.05). Western blots for each set of reference protein and target protein were from one blot, and the black line was the cropped edge of the blot.

By examining the mTOR pathway in SCs, we found no significant difference in mTOR protein expression was found among the four groups (*p* > 0.05, Figure 3A). The level of mTOR phosphorylation (P–mTOR) was significantly higher in the 0.80% Lys/0.40% Met composition than in the other three groups (*p* < 0.05, Figure 3B). Similarly, the 0.80% Lys/0.40% Met composition group displayed the largest P–mTOR/mTOR ratio of the three groups, which differed significantly from that of the group receiving the 1.00% Lys/0.40% Met composition level (*p* < 0.05, Figure 3C).

**Figure 4:**
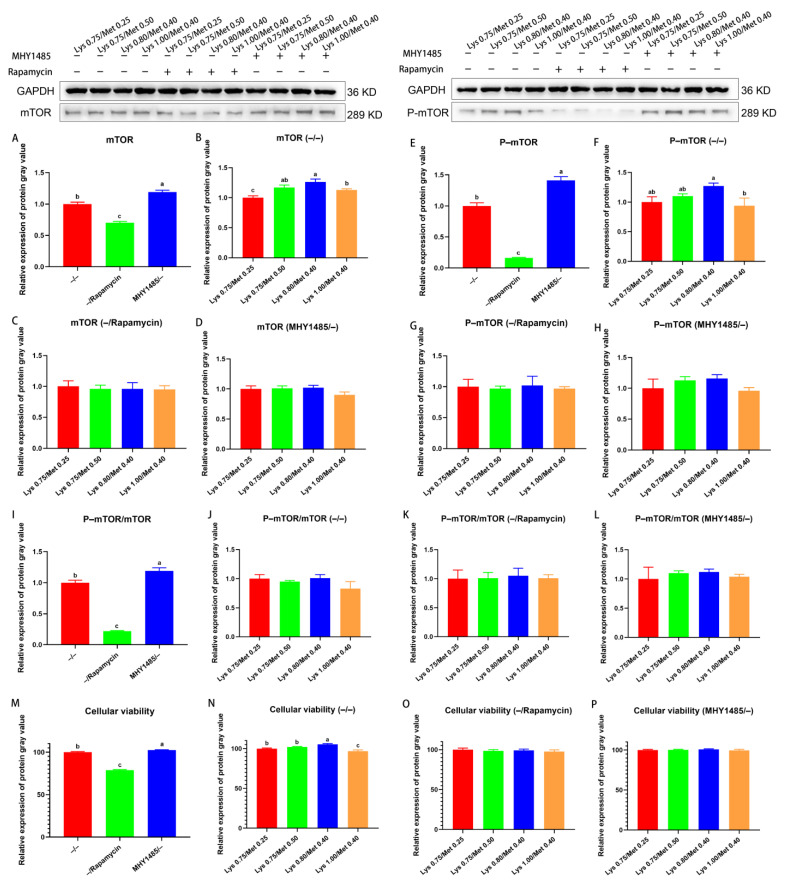
Effects of different lysine and methionine composition on mTOR signaling pathway and cell viability in SCs. MHY1485 (mTOR signaling pathway activator; −: no MHY1485 added, +: MHY1485 added), rapamycin (mTOR signaling pathway inhibitor; −: no rapamycin added, +: rapamycin added). −/−: no MHY1485 added and no rapamycin added; −/Rapamycin: no MHY1485 added and rapamycin added; MHY1485/−: MHY1485 added and no rapamycin added. Detection of mTOR signaling pathway activation or inhibition in SCs (**A**). The expression of mTOR signaling pathway in the four experimental groups after SCs were treated differently by −/− (**B**), −/Rapamycin (**C**), and MHY1485/− (**D**), respectively. Detection of P–mTOR signaling pathway activation or inhibition in SCs (**E**). The expression of P–mTOR signaling pathway in the four experimental groups after SCs were treated differently by −/− (**F**), −/Rapamycin (**G**), and MHY1485/− (**H**), respectively. Ratio of phosphorylated protein to total protein (**I**–**L**). I = E/A; J = F/B; K = G/C; L = H/D. Cell viability assay after activation or inhibition of mTOR signaling pathway in SCs (**M**). Cell viability assay in four experimental groups after different treatments of SCs with −/− (**N**), −/− rapamycin (**O**), and MHY1485/− (**P**), respectively. Data are expressed as means ± SEM (*n* = 3). Comparisons between groups that contain only different lowercase letters indicate significant differences (*p* < 0.05). Western blots for each set of reference protein and target protein were from one blot, and the black line was the cropped edge of the blot.

Further, mTOR protein expression in cells treated with −/rapamycin was significantly lower than that in cells treated with −/− or MHY1485/− (*p* < 0.05, Figure 4A). In the −/− treated cells, mTOR protein expression was significantly higher in the 0.80% Lys/0.40% Met composition group than in that containing 0.75% Lys/0.25% Met and 1.00% Lys/0.40% Met (*p* < 0.05, Figure 4B). No significant difference in mTOR protein expression was observed among the four groups of cells treated with −/rapamycin or MHY1485/− (*p* > 0.05, Figure 4C,D). The levels of P–mTOR were significantly downregulated in cells treated with −/rapamycin compared with that in cells treated with −/− or MHY1485/− (*p* < 0.05, Figure 4E). In cells treated with −/−, P–mTOR levels were significantly higher in the 0.80% Lys/0.40% Met composition group than in the group administered 1.00% Lys and 0.40% Met (*p* < 0.05, Figure 4F). No significant difference in P–mTOR protein expression was observed among the four groups of cells treated with −/rapamycin or MHY1485/− (*p* > 0.05, Figure 4G,H). We further found that the P–mTOR/mTOR ratio was significantly smaller in −/rapamycin–treated cells than in cells treated with −/− or MHY1485/− (*p* < 0.05, Figure 4I). There was no significant difference in the P–mTOR/mTOR ratio among the respective internal four test groups treated with −/−, −/rapamycin, or MHY1485/− (*p* > 0.05, Figure 4J–L). Viability was significantly reduced in cells treated with −/rapamycin compared with that in cells treated with −/− or MHY1485/− (*p* < 0.05, Figure 4M). Among the four groups treated with −/−, cell viability was significantly higher in the 0.80% Lys/0.40% Met composition group than the other three groups (*p* < 0.05, Figure 4N). No significant difference in cell viability was recorded between their respective four experimental groups treated with −/rapamycin or MHY1485/− (*p* > 0.05, Figure 4O,P).

### 3.5. Effect of SLC38A on mTOR Signaling Pathway

**Figure 5:**
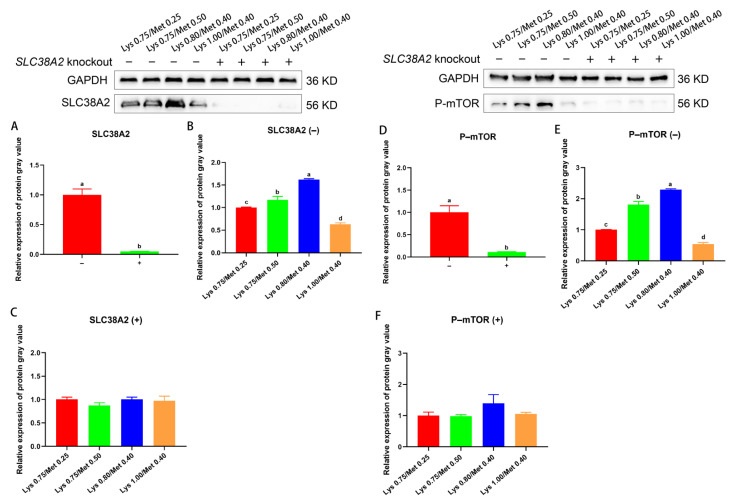
Effects of different lysine and methionine composition on SLC38A2–mTOR signaling pathway in SCs. Protein expression of SLC38A2 knockout assay in SCs (−: SLC38A2 gene not knocked out; +: *SLC38A2* gene knocked out). Knockout check of *SLC38A2* in SCs (**A**); Expression of SLC38A2 in SCs without (**B**) or after *SLC38A2* knockout (**C**) was detected in each of the four experimental groups. Expression of P–mTOR in SCs without or after knockout of *SLC38A2* (**D**). Expression of P–mTOR was detected in four experimental groups without (**E**) or after knockout (**F**) of *SLC38A2*, respectively. Data are expressed as means ± SEM (*n* = 3). Comparisons between groups that contain only different lowercase letters indicate significant differences (*p* < 0.05). Western blots for each set of reference protein and target protein were from one blot, and the black line was the cropped edge of the blot.

To verify the upstream signaling role of SLC38A2, we performed *SLC38A2* knockout assays on SCs and found the SLC38A2 protein expression was significantly reduced in the knockout group (*p* < 0.05, Figure 5A). Meanwhile, P–mTOR protein expression was significantly downregulated in the *SLC38A2* knockout group compared with the *SLC38A2* non-knockout group (*p* < 0.05, Figure 5D). In the SCs after *SLC38A2* non-knockout, both SLC38A2 and P–mTOR protein expression were highest in the 0.80% Lys/0.40% Met composition group (*p* < 0.05, Figure 5B,E). However, in SCs after *SLC38A2* knockout, SLC38A2 and P–mTOR protein expression were not significantly different in any of the four experimental groups (*p* > 0.05, Figure 5C,F).

## 4. Discussion

Lys and Met are essential amino acids for the nutritional needs of monogastric animals and play many important metabolic functions. Appropriate Lys and Met intake is important to ensure healthy growth, development, and reproduction [21,22]. Early studies in pigs found that diets containing 1.8% Lys and 0.50% Met could greatly improve performance regarding average daily gain and average daily feed intake [23]. Limited increases in Lys and Met concentrations in broiler diets can improve feed conversion, body weight, carcass yield, and breast muscle production [24]. In rabbits, meanwhile, dietary Lys and Met supplementation was reported to not be effective at lowering the incidence of enteritis in rabbits, but led to a significant increase in the weaning weight of young animals [25]. Similarly, in this study, we found that diets containing the 0.80% Lys/0.40% Met composition promoted the greatest average daily gain in rabbits and also significantly improved feed conversion.

During or after nutritional intake, amino acid homeostasis is primarily controlled via autoregulatory processes. Amino acid transporters play a crucial role in the distribution and circulation of amino acids in cells and organs [26]. In our study, we found that the expression of genes encoding amino acid transporter proteins, namely SLC7A10 (ASC–1) and SLC38A2, were significantly upregulated in the muscle of rabbits in the 0.80% Lys/0.40% Met composition group. SLC7A10 is mainly involved in mediating the Na+–independent transport of glycine, L–alanine, L–cysteine, and other amino acids [27]. SLC38A2 is a system A transporter that accumulates small neutral amino acids directly or indirectly through the activation of the ASCT1 and LAT1/2 transporter proteins [28]. These indicated that the different compositions of lysine and methionine in the diet could affect the absorption of other amino acids. Our experimental results also found that the 0.80% Lys/0.40% Met composition group reduced nitrogen emissions and increased the efficiency of nitrogen utilization in rabbits.

Plasma urea, a product of hepatic nitrogen metabolism, is negatively correlated with protein utilization. Amino acid balance is essential for improving protein utilization and reducing plasma urea levels [29]. In this study, we found that rabbits fed diets containing 0.80% Lys and 0.40% Met had the lowest plasma urea and uric acid contents and the highest nitrogen utilization efficiency. This suggests that reasonable levels of Lys and Met composition (0.80% Lys and 0.40% Met) in the diet can promote amino acid balance, which was in agreement with that previously reported [30,31,32].

The supplementation of limiting amino acids to the diet, either alone or in combination with other nutrients, is a feasible approach for improving animal production performance [33]. Improving dietary-restrictive amino acid content can reportedly improve chicken breast tenderness and carcass yield, and, to some extent, also muscle quality [34]. In addition, Lys supplementation was shown to increase sarcoplasmic protein concentrations, an effect that was positively correlated with muscle tenderness [35]. The characteristics of muscle fibers are important determinants of the quality of meat and are closely related to traits such as color, tenderness, pH, and water retention properties [36]. Based on contractile and metabolic properties, muscle fibers are usually classified as oxidative/slow (type I) or glycolytic/rapid (type II) [37]. Type I muscle fibers have been positively associated with good meat quality, while a greater percentage of type II fibers was negatively correlated with a higher incidence of pale, soft, and exuding (PSE) meat [38]. In general, the initial pH of rabbits at 45 min post-mortem is approximately between 6.1 and 6.9, and the shear force of rabbit muscle is approximately 11.45 N [39,40]. After 24 h, the pH of rabbit meat varied between approximately 5.66 and 5.80 [40]. Consistent with previous reports, we found that as the Lys content in the diet increased, the proportion of type I muscle fibers in skeletal muscle increased, muscle tenderness increased, and muscle water retention capacity was enhanced, thereby improving muscle quality to some extent.

Growth in the number and size of muscle fibers determines the main process of muscle growth [41]. In developing muscle, SCs undergo extensive proliferation and most of them fuse with muscle fibers; in contrast, SCs may undergo apoptosis during muscle atrophy [42]. For high developmental growth, new fibers are formed on the surface of existing fibers by fusion of satellite cells to form multinucleated myotubes [43]. In the present study, it was found that the 0.80% Lys and 0.40% Met group had the lowest apoptosis rate and increased cell division and cell migration ability. This indicated that the presence of 0.80% Lys and 0.40% Met in the diet could, to some extent, prevent muscle atrophy, promote satellite cell division and proliferation, and maintain normal muscle condition. Muscle hyperplasia and hypertrophy involve populations of myogenic precursor cells, which are also called satellite cells and are regulated by a number of both positive and negative factors. MYOD, Myf5, and MYOG are involved in the positive regulation of muscle development, while MSTN has a negative effect on muscle development [44]. Further, the combination of our results showed the involvement of Myf5 and MYOG in the positive regulation of 0.80% Lys and 0.40% Met composition on the promotion of muscle development.

It is also reported that SLC38A2 is extensively regulated by cellular stress, nutritional availability, and hormonal signaling, and acts as an amino acid sensor upstream of mTOR in the regulation of processes such as protein synthesis and cell proliferation [45]. Interestingly, we have now also found a direct involvement of SLC38A2 in regulating the effect of 0.80% Lys and 0.40% Met on the mTOR signaling pathway. Additionally, the combined results showed that 0.80% Lys and 0.40% Met increased SCs viability via the SLC38A2–mTOR signaling pathway, which in turn promoted the growth and development of muscle tissue.

## 5. Conclusions

In all test diets, 0.80% Lys/0.40% Met was the most suitable lysine and methionine composition. Furthermore, 0.80% Lys/0.40% Met promoted the absorption and utilization of dietary nitrogen by rabbits, and adjusted the growth status and production performance of rabbits, especially promoting muscle growth, development, and muscle quality. SLC38A2 acted as an amino acid sensor upstream of mTOR and was involved in the 0.80% Lys/0.40% Met regulation of muscle growth and development, thus implicating the mTOR signaling pathway in these processes.

## Figures and Tables

**Table 1:** Effects of different lysine and methionine composition in the diet on the production performance of rabbits (*n* = 30).

Item	Lys 0.75/Met 0.25	Lys 0.75/Met 0.50	Lys 0.80/Met 0.40	Lys 1.00/Met 0.40	SEM	*p*-Values
ADFI (g)	135.46 ^a^	123.84 ^b^	124.70 ^b^	117.32 ^c^	1.67	<0.0001
ADG (g)	39.85 ^b^	41.47 ^ab^	43.90 ^a^	38.75 ^b^	0.94	0.0012
Feed conversion ratio (%)	29.42 ^c^	33.49 ^b^	35.20 ^a^	33.03 ^bc^	0.83	<0.0001
Liver weight/Live weight (%)	2.58	2.65	2.69	2.68	0.12	0.9045
Kidney weight/Live weight (%)	0.55 ^b^	0.62 ^a^	0.60 ^ab^	0.59 ^ab^	0.02	0.0661

Abbreviations: ADFI = average daily feed intake; ADG = average daily gain. Means without a common lowercase superscript letter in a row are different in *p* < 0.05.

**Table 2:** Effect of different lysine and methionine composition in diets on nitrogen metabolism in rabbits (*n* = 6).

Item	Lys 0.75/Met 0.25	Lys 0.75/Met 0.50	Lys 0.80/Met 0.40	Lys 1.00/Met 0.40	SEM	*p*-Values
Intake of nitrogen (g/d)	4.78	4.74	4.63	4.69	0.09	0.6914
Nitrogen in feces (g/d)	1.47 ^a^	1.30 ^b^	1.18 ^b^	1.28 ^b^	0.05	0.0108
Urinary nitrogen (g/d)	1.63 ^a^	1.49 ^ab^	1.38 ^b^	1.64 ^a^	0.07	0.0215
Digestible nitrogen (g/d)	3.30	3.44	3.45	3.40	0.10	0.7258
Retention nitrogen (g/d)	1.67	1.95	2.08	1.76	0.13	0.1964
NAD (%)	69.10 ^b^	72.48 ^ab^	74.48 ^a^	72.62 ^ab^	1.22	0.0432
NU (%)	34.56 ^b^	40.92 ^ab^	44.66 ^a^	37.52 ^ab^	2.28	0.0457
NBV (%)	50.24 ^b^	56.25 ^ab^	59.82 ^a^	51.67 ^ab^	2.57	0.0736

Abbreviations: NAD = nitrogen apparent digestibility; NU = nitrogen utilization; NBV = nitrogen biological value. Means without a common lowercase superscript letter in a row are different in *p* < 0.05.

**Table 3:** Effect of different lysine and methionine composition in diets on blood biochemical indices in rabbits (*n* = 6).

Item	Lys 0.75/Met 0.25	Lys 0.75/Met 0.50	Lys 0.80/Met 0.40	Lys 1.00/Met 0.40	SEM	*p*-Values
Albumin (g/L)	33.78	34.02	33.12	34.75	1.03	0.7465
Glucose (mmol/L)	6.82	6.79	6.61	6.85	0.15	0.7009
Total cholesterol (mmol/L)	1.42	1.53	1.74	1.91	0.20	0.3588
Triglyceride (mmol/L)	1.01	1.12	1.02	1.30	0.21	0.7824
Total protein (g/L)	63.47	67.63	67.73	63.07	1.95	0.2360
Uric acid (μmol/L)	12.17 ^ab^	10.67 ^ab^	8.83 ^b^	16.17 ^a^	1.75	0.0608
Urea (mmol/L)	8.60 ^ab^	7.31 ^b^	7.27 ^b^	8.94 ^a^	0.44	0.0361

Means without a common lowercase superscript letter in a row are different in *p* < 0.05.

**Table 4:** Effect of different lysine and methionine composition in diets on muscle quality in rabbits (*n* = 6).

Item	Lys 0.75/Met 0.25	Lys 0.75/Met 0.50	Lys 0.80/Met 0.40	Lys 1.00/Met 0.40	SEM	*p*-Values
Muscle shear force (N)	14.80 ^a^	11.26 ^bc^	11.63 ^b^	10.16^c^	0.61	0.0026
Drip loss (%)	11.25 ^ab^	11.75 ^a^	7.63 ^ab^	7.13 ^b^	1.30	0.0460
Flesh color (a *)	5.86	6.53	6.48	4.58	0.70	0.2215
Flesh color (b *)	7.39	7.1	7.15	6.94	0.36	0.8519
Flesh color (L *)	38.33	38.90	36.60	39.65	1.08	0.2740
Muscle pH (45 min)	6.32 ^a^	6.16 ^ab^	6.06 ^b^	6.11 ^ab^	0.08	0.0615
Muscle pH (24 h)	5.83	5.78	5.72	5.78	0.11	0.5062

Means without a common lowercase superscript letter in a row are different in *p* < 0.05 (muscles measured in the table are the dorsolumbar muscles, more specific information is available in the Supplementary Materials).

## Data Availability

None of the data were deposited in an official repository. The data presented in this study are available on request from the corresponding author.

## Supplementary Materials

The following supporting information can be downloaded at: https://www.mdpi.com/article/10.3390/ani12233406/s1, Figure S1: Effects of different lysine and methionine composition of diets on the growth of rabbits in Exp. 1; Figure S2: Identification of isolated primary muscle satellite cells; Supplementary Table S1: Composition and nutrient levels of basal diet (air–dry basis); Supplementary Table S2: The amount of amino acids added at the nutritional level of basal diet in different treatment groups (g/kg); Supplementary Table S3: Gene-specific primers used for the analysis of rabbit gene expression; Supplementary Table S4: Contents of free amino acids in plasma (μg/mL); Supplementary Materials and methods.
