# Supplementary material for: Effects of the SLC38A2–mTOR Pathway Involved in Regulating the Different Compositions of Dietary Essential Amino Acids–Lysine and Methionine on Growth and Muscle Quality in Rabbits
Source: Animals (Basel). 2022 Dec 3;12(23):3406. doi: 10.3390/ani12233406 (PMC9740809; doi:10.3390/ani12233406)
Supplement: Supplementary file 1 [file animals-12-03406-s001.zip › animals-2066165-supplementary.pdf]

Supplementary materials

Effects of the SLC38A2-mTOR pathway involved in regulating the different composition of dietary essential amino acids-lysine and methionine on growth and muscle quality in rabbits

Bin Zhang [1], Boyuan Ning [1], Xiaoyang Chen [1,2,3], Chenyang Li [1,2,4], Mengqi Liu [1], Zhengkai Yue [1], Lei Liu [1*] and Fuchang Li [1*]

[1] Key Laboratory of Efficient Utilization of Non-Grain Feed Resources (Co-Construction by Ministry and Province), Ministry of Agriculture and Rural Affairs, Shandong Provincial Key Laboratory of Animal Biotechnology and Disease Control and Prevention, Department of Animal Science, Shandong Agricultural University, Taian 271018, China

[2] State Key Laboratory of Animal Nutrition, Institute of Animal Science, Chinese Academy of Agricultural Sciences, Beijing 100193, China

[3] College of Animal Science and Technology, Northwest A&F University, Shanxi 712100, China

[4] College of Animal Science and Technology, China Agricultural University, Beijing 100083, China

* Correspondence: leiliu@sdau.edu.cn (L.L.); chlf@sdau.edu.cn (F.L.)

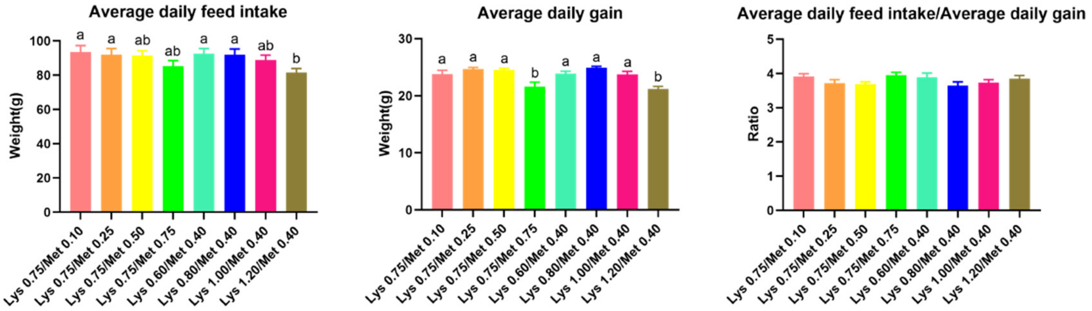

Figure S1. Effects of different lysine and methionine composition of diets on the growth of rabbits in Exp. 1. Date are expressed as mean ± SEM, n = 30. Comparisons between groups that contain only different lowercase letters indicate significant differences ($P < 0.05$).

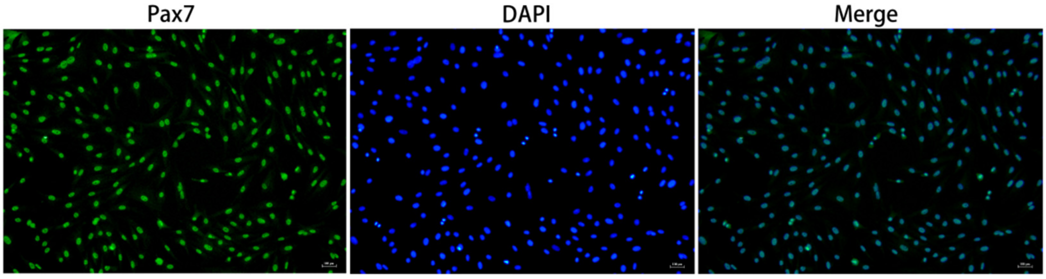

Figure S2. Identification of isolated primary muscle satellite cells. Pax7: paired box 7.

Supplementary Table S1. Composition and nutrient levels of basal diet (air-dry basis)

Ingredients	Content (%)	Nutrient levels[2]	Content
Corn	8.2	DE(MJ/kg)	9.47
Wheat middlings	5	CP (%)	15.65
Soybean meal	6	EE (%)	3.81
Corn germ meal	12	CF (%)	19.35
Wheat bran	18	Ca (%)	0.73
Sunflower meal	11.5	P (%)	0.55
Soybean oil	1.3	Lys (%)	0.41
Alfalfa meal	15	Met (%)	0.13
Peanut shell powder	13	Arg (%)	0.21
Rice husk powder	6	Cys (%)	0.22
Premix[1]	4	Thr (%)	0.58
Total	100	His (%)	0.32

[1]The premix provided the following per kg of diets: Salt 5 g, Calcium hydrogen phosphate 6 g, Choline (50 %) 120 mg, Compound mineral 2 g, Compound vitamin 0.3 g, Baking soda 0.5 g, sweeteners 50 mg. The rest was miscellaneous meal carrier complement.

[2]Nutrient levels were measured values. DE=Digestible Energy; DM=Dry Matter; CP=Crude Protein; EE = Ether extract; CF = Crude Fiber; Ca = Calcium; P = Phosphorus; Lys = Lysine; Met = Methionine; Arg= Arginine; Cys = Cystine; Thr = Threonine; His = Histidine.

Supplementary Table S2. The amount of amino acids added at the nutritional level of basal diet in different treatment groups (g/kg)

	**Lys 70%**	**Met 99%**
Lys 0.75/Met 0.25	5.29	1.2
Lys 0.75/Met 0.50	5.29	3.7
Lys 0.80/Met 0.40	5.57	2.7
Lys 1.00/Met 0.40	8.34	2.7

Supplementary Table S3. Gene-specific primers used for the analysis of rabbit gene expression.

**Gene**	**Genebank accession number.**	**Primers sequences(5′→3′)**	**Product size (bp)**
β-actin	NM_001101683.1	F: CGCAGAAACGAGACGAGATT<br>R: GCAGAACTTTGGGGACTTTG	168
GAPDH	NM_001082253	F: TGCCACCCACTCCTCTACCTTCG<br>R: CCGGTGGTTTGAGGGCTCTTACT	163
MSTN	NM_001109821.1	F: TCAAGGTAACGGACACACCA<br>R: CGCAATAATCCAATCCCATC	137
Myf5	NM_001319577.1	F: TTAACCAGGCTTTCGAGAC<br>R: TCTCCACCTGCTCTCTCAGC	135
MYOD	XM_008275114.2	F: GAAGCACTTACAAGCGGCGACTC<br>R: ACAGGCAGTCAAGGCTGGACAC	190
MYOG	XM_002717584.3	F: TGTGTAAGAGGAAGTCAGTGTC<br>R: CGCTCGATGTACTGGATGG	178
SLC7A2	XM_008251103.2	F: CGCAACAACTGGTGAGGAAG<br>R: CTCCGCCATAGCATAGATTACAC	286
SLC7A5	NM_001082120.1	F: CCTCTGTCCTGTCCATGATCCA<br>R: AAGAAGTTGATGACGGAGAAGATGT	121
SLC7A8	NM_001082682.1	F: GCTTCATCAACTACCTCTTCTAC<br>R: ATACACAACCACGCACATCT	311
SLC710	XM_008257285.2	F: TGGCTACCTGTTCACCTCCTC<br>R: CGTAGTTGATGAGCGTGTATGTG	179
SLC38A2	XM_002712695.3	F: CACACCTACTCCACCGTCAT<br>R: TGCCAAGATAGACACAGTGATG	189

GAPDH: glyceraldehyde 3-phosphate dehydrogenase; *MSTN*: myostatin; *Myf5:* myogenic factor 5; *MYOD:* myogenic differentiation; *MYOG:* myogenin; *SLC7A2:* solute carrier family 7 member 2; *SLC7A5:* solute carrier family 7 member 5; *SLC7A8:* solute carrier family 7 member 8; *SLC710:* solute carrier family 7 member 10; *SLC38A2:* solute carrier family 38 member 2

Supplementary Table S4. Contents of free amino acids in plasma (μg/mL)

	**Lys (plasma)**	**Met (plasma)**
Lys 0.75/Met 0.25	94.74	21.64
Lys 0.75/Met 0.50	86.83	39.89
Lys 0.80/Met 0.40	128.55	34.85
Lys 1.00/Met 0.40	131.30	29.08

Materials and methods

Detection of muscle quality

Flesh color: Within 45 min after slaughter, the flesh color of the dorsal lumbar muscle of the fourth thoracic vertebra, the dorsal lumbar muscle of the eighth thoracic vertebra, and the dorsal lumbar muscle of the third lumbar vertebra were evaluated in each animal. An incision was made at the inspection site and the flesh color was assessed with a Konica Minolta CR-10 colorimeter using the CIE color system (L*: lightness, a*: redness, b*: yellowness). Values were determined at three locations along the loin (anterior, mid, and posterior part) from the upper dorsal back and the average of three test values was recorded. Muscle shearing force: A meat sample (1 cm^3) was obtained from the same site where the meat color test was performed. Testing was carried out using the C-LM3B Tenderness Meter (TENOVO, China) and the average of the three test values was recorded. Muscle pH: Muscle pH was measured at the same site where the meat color test was performed using a pH meter (SevenExcellence PH meter S400, Mettler Toledo, USA). Measurements were taken at 45 min and 24 h after slaughter. The average of three test values was recorded. Drip loss: A 1 cm3 piece of muscle was removed from the tenth thoracic vertebra of the dorsal lumbar muscle following which the muscle was immediately weighed (Initial weight A). The muscle was then sealed in a plastic pouch, which was hung by a thread at atmospheric pressure at 4 °C. The muscle was reweighed after 24 h (Weight B). Drip loss = (Initial weight A − Weight B)/Initial weight A.

Plasma biochemistry

Plasma albumin (AL), total protein (TP), urea, uric acid (UA), glucose (GLU), triglyceride (TG), and total cholesterol (TCHO) contents were measured using an automatic biochemical analyzer (HITACHI 7020; Hitachi, Japan).

Immunofluorescence

Paraffin sectioning: Vertical muscle fibers from fresh hind leg muscle were sampled to a thickness of approximately 0.5 cm and fixed in 4 % paraformaldehyde for 24 h. The fixed tissue was removed from the fixative, trimmed flat, labeled, and placed in a dehydration box. The dehydration box was subsequently placed in a dehydrator (Donatello, DIAPATH) for dehydration using a graded alcohol series. The protocol included 75 % alcohol for 4 h, 85 % alcohol for 2 h, 90 % alcohol for 2 h, 95 % alcohol for 1 h, anhydrous ethanol I for 30 min, anhydrous ethanol II for 30 min, alcohol benzene for 5–10 min, xylene I for 5–10 min, xylene II for 5–10 min, melted paraffin wax I (65 °C) for 1 h, melted paraffin wax II (65 °C) for 1 h, and melted paraffin wax III (65 °C) for 1 h. The wax-soaked tissue was embedded in an embedding machine (JB-P5, Wuhan Junjie Electronics Co., Ltd, Wuhan, China). Once the wax had solidified, the wax block was removed from the embedding frame, trimmed, cooled to −20 °C, and cut into 4 μm sections using a pathology slicer (RM2016, Shanghai Leica Instrument Co., Ltd, Shanghai, China).

Paraffin section immunofluorescence: The sections were rehydrated by placing in xylene I for 15 min, xylene II for 15 min, anhydrous ethanol I for 5 min, anhydrous ethanol II for 5 min, 85 % alcohol for 5 min, and 75 % alcohol for 5 min, followed by washing with distilled water. Antigen retrieval was performed in a microwave oven in EDTA antigen repair buffer (pH 8.0, AM9912, ThermoFisher, Shanghai, China). Tissue sections were then placed in a 3 % hydrogen peroxide solution and incubated for 25 min at room temperature, protected from light, to block endogenous peroxidases, and then washed three times (5 min each wash) in PBS (pH 7.4) on a decoloring shaker. After shaking off the PBS, the tissue samples were blocked in 3 % BSA(36101ES25, Yeasen Biotechnology, Shanghai, China) (added dropwise) for 30 min, and incubated overnight at 4 °C in a wet box with a drop of the first primary antibody diluted in PBS. After three 5-min washes in PBS (pH 7.4) on a decoloring shaker, each section was incubated with a drop of the appropriate HRP-labeled secondary antibody for 50 min at room temperature, with shaking. The slides were washed three times with shaking in PBS (pH 7.4) on a decoloring shaker, 5 min each wash. The sections were then lightly shaken dry and incubated for 10 min at room temperature, protected from light, with a drop of BSA, and

subsequently washed three times, 5 min each wash, on a decoloring shaker in TBST (PS103, Epizyme Biomedical, Shanghai, Chian). The tissue sections were again subjected to antigen retrieval by placing them in EDTA antigen retrieval buffer (pH 8.0) and heating in a microwave for 8 min on medium heat and 8 min on ceasefire to 7 min on medium-low heat to remove the bound primary and secondary antibodies. A second primary antibody in PBS was then added dropwise to the sections, which were then incubated flat in a wet box at 4 °C overnight. The slides were washed three times in PBS (pH 7.4) on a decoloring shaker, 5 min each wash. The sections were lightly shaken dry, covered with a drop of the corresponding fluorescent secondary antibody, and incubated for 50 min at room temperature, protected from light. The slices were dried with light shaking, incubated for 10 min at room temperature in the dark with DAPI staining solution, and washed three times (5 min each wash) with PBS (pH 7.4) on a decoloring shaker. After lightly shaking dry, an autofluorescence quencher was added to the circle for 5 min and the sections were rinsed in running water for 10 min. The slices were lightly shaken and sealed with an anti-fluorescence quenching agent. Sections were observed and imaged under a fluorescence microscope.

SCs isolation and culture

Two-day-old rabbits were euthanized by neck dislocation. Part of the muscle of the hindlimb was isolated under aseptic conditions, placed in a sterile Petri dish, and rinsed with 70 % alcohol for 1 min. The sample was then washed twice with phosphate-buffered saline (PBS; P1020, Solarbio, China) and the fascia, blood vessels, and adipose tissue were removed as much as possible. The muscle was cut into small pieces (1 mm^3) with ophthalmic scissors, rinsed with PBS, and left to stand for 1 min. After discarding the upper liquid and floating tissue, 0.25 % trypsin digestion solution (without phenol red or EDTA (T1350, Solarbio) was added to the sample, and digestion was allowed to proceed at 37 ℃ for 20 min. Growth medium (89 % DMEM/F12, 10 % fetal bovine serum [FBS], 1 % penicillin–streptomycin; Thermo Fisher) was then added to stop the digestion and the solution was pipetted repeatedly to separate the cells. The solution was then filtered through a 38-μm-pore-size filter screen into a centrifuge tube and centrifuged at 1,000 rpm for 5 min. After discarding the supernatant, the cells were washed twice with PBS, suspended in culture medium

supplemented with 20 % FBS, inoculated in a culture flask without lysine treatment, and cultured for 60 min. Then, the upper liquid was reinoculated into a clean culture bottle without lysine treatment, and culture medium containing 20 % FBS was added, after which the cells were cultured for another 60 min. The upper liquid was collected for counting using trypan blue and the cells were inoculated into polylysine-treated culture bottles. The growth status and morphology of the cells were observed and the culture medium was changed after 48 h. At 80 % confluence, the cells were detached with 0.25 % trypsin and passaged at a split ratio of 1:2. The culture medium was changed every 24 h and the growth state was observed under an inverted microscope.

SCs identification

Remove the cell crawl from the medium when the cells had reached 85 % confluence. The cells were washed three times with cool PBS, 5 min each wash, fixed in methanol at 4 °C for 20 min, washed three times with PBS (5 min each wash), and permeabilized with 0.5 % Triton X-100 for 10 min. After three 5 min PBS washes, the cells were blocked at room temperature for 20 min, dried, and then incubated with primary antibody overnight at 4 °C. After washing three times with PBS, 5 min each wash, the cells were incubated with suitably diluted biotin-labeled anti-mouse IgG antibody (Bioss, Beijing, China) at 37 °C for 30 min, washed with PBS (3 times, 5 min each wash), incubated with diluted SABC-FITC (Solarbio, Beijing, China) at 37 °C for 30 min, and washed again with PBS (4 × 5 min). Finally, the cells were sealed with an anti-fluorescence quenching agent and observed and imaged under a fluorescence microscope. The identification results are shown in Figure S2.

The cell migration assay

The cells were passaged to four 6-well plates (one for each treatment group). After using the previous growth medium, the fusion degree was about 80 %, and then the medium was starved with PBS for 24 h. DMEM/F12 without lysine and methionine was used as the basic medium (Boster, China). According to the plasma lysine and methionine contents of animal experiment (Table S4), the amounts of lysine and methionine added to the culture medium were as follows: (1) 94.74 μg/mL Lys and 21.64 μg/mL Met; (2) 86.83 μg/mL Lys and 39.89

μg/mL Met; (3) 128.55 μg/mL Lys and 34.85 μg/mL Met; and (4) 131.3 μg/mL Lys and 29.08 μg/mL Met. A 1000 μL pipette tip was used to make vertical scratches in the culture plate while ensuring that each scratch was as wide as possible. Four 6 well plates were added with four different lysine and methionine contents of the medium. Cell growth was observed under an inverted microscope at 4, 8, 16, and 24 h.

The cell cycle and apoptosis assays

Cell cycle analysis: Cells were washed with cold PBS and centrifuged at 1,000 rpm for 10 min. After discarding the supernatant, 75 % alcohol was added to the cells dropwise, following which the cells were incubated at −20 °C for 18 h, centrifuged at 1,000 rpm for 10 min, and the supernatant was discarded. Cell apoptosis: Cells were washed twice with cold PBS and then resuspended in 1 x Binding buffer at a concentration of 1×10^6 cells/mL. Take 100 μL (1×10^5 cells)of the Binding buffer solution and transfer it to a 1.5 mL opaque centrifuge tube. Negative control: 100 μL (1×10^5 cells)of the Binding buffer solution + 400 μL 1 x Binding buffer; Single positive 1 (AV-FITC): 100 μL (1×10^5 cells)of the Binding buffer solution + 400 μL 1 x Binding buffer + 5 μL AV-FITC; Single positive 2 (PI): 100 μL (1×10^5 cells) of the Binding buffer solution + 400 μL 1 x Binding buffer + 5 μL PI; Test samples (AV-FITC+PI): 100 μL (1×10^5 cells) of the Binding buffer solution + 400 μL 1 x Binding buffer + 5 μL AV-FITC + 5 μL PI. Incubated at 25°C for 15 minutes in the dark and assay using BD fortessa flow cytometer.

mTOR pathway activation and inhibition assays

SCs were treated with four different concentrations of lysine and methionine containing MYH1485 (10 μM) or rapamycin (100 nM) medium for cell viability and mTOR Western blotting, respectively.
